# Phase 1 pilot study of RRx-001 + nivolumab in patients with advanced metastatic cancer (PRIMETIME)

**DOI:** 10.3389/fimmu.2023.1104753

**Published:** 2023-03-07

**Authors:** Tony Reid, Bryan Oronsky, Scott Caroen, Mary Quinn, Jeannie Williams, Pedro Cabrales, Nacer Abrouk

**Affiliations:** ^1^ Department of Bioengineering, University of California at San Diego, San Diego, CA, United States; ^2^ EpicentRx, Torrey Pines, CA, United States; ^3^ Clinical Trial Innovations, Mountain View, CA, United States

**Keywords:** RRx-001, CD-47, tumor associated macrophage, vascular normalization, nivolumab, cold tumors, NLRP3 inflammasome inhibitor, bromonitrozidine

## Abstract

**Background:**

Bromonitrozidine (RRx-001) is a minimally toxic, NLRP3 inhibitor that has been observed, in experimental systems, to also downregulate CD47, repolarize tumor associated macrophages (TAMs) and normalize aberrant tumor perfusion. This phase 1 pilot study was undertaken to determine the safety and feasibility of RRx-001 and nivolumab in patients with advanced cancer and no standard options.

**Methods:**

This single arm, single site, open-label pilot study (NCT02518958) called PRIMETIME was designed to evaluate the safety profile of RRx-001 and nivolumab in patients with advanced malignancies and no other standard therapeutic options. A 3 + 3 trial design was used to establish safety of the combination at each dose level and guide the decision to escalate dose. RRx-001 is infused once weekly while nivolumab is given at 3mg/kg once every 2 weeks. The RRx-001 starting dose was 2 mg IV weekly with 4 dose level escalations up to 16 mg IV weekly. From January 2015 to November 2015, twelve patients received treatment for only 4 cycles (total 12 weeks) with the combination due to unavailability of nivolumab, which was not supplied to the Sponsor. Treatment-emergent (all cause, TEAEs) and treatment-related (TRAEs) adverse events that occurred within 16 weeks of the first dose of RRx-001 and nivolumab were characterized according to CTCAE v4.03.

**Results:**

Twelve patients received ≥1 dose of RRx-001 and nivolumab. One discontinuation occurred due to pneumonitis and one to voluntary withdrawal after a post-procedural infection. There were no DLTs. The main adverse event related to RRx-001 was infusion reaction (33.3%). The main adverse event related to the combination was pseudoprogression manifested by larger tumors in patients that were symptomatically improved (25%). The most common immune-related treatment-emergent AEs were pneumonitis (8.3%), and hypothyroidism (8.3%). The objective response rate at 12 weeks was 25% and the disease control rate (DCR) consisting of ≥SD was 67% by Response Evaluation Criteria in Solid Tumors (RECIST) version 1.1. 25% of the patients progressed on the combination.

**Conclusions:**

The combination of RRx-001 and nivolumab was safe and well-tolerated with preliminary evidence of anti-cancer activity. Further clinical trials with RRx-001 and nivolumab are warranted.

**Clinical trial registration:**

ClinicalTrials.gov identifier, NCT02518958.

## Introduction

Checkpoint inhibitors (CIs) such as anti-PD-1, anti-PD-L1 and anti-CTLA-4 have demonstrated activity in multiple immunogenic tumor types, including melanoma, NSCLC, SCLC, bladder, renal, and head and neck (1) However, many patients do not benefit (less than 30% of the patients with so-called immunogenic tumors are responsive and about a quarter of the responsive patients develop resistance (2)) due to several factors such as the co-optation of other inhibitory pathways like TIM3, LAG-3 and IDO or the release of pro-tumoral, immunosuppressive cytokines such as TGF-β or IL-10 that collectively induce tolerance (3).

One strategy that has been tried to increase the cytotoxic activity of CD8 T cells and to overcome the immunosuppressiveness of the tumor microenvironment is the administration of immunotherapy in combination: checkpoint inhibitors with non-overlapping mechanisms of action such as an anti-PD-1 and an anti-CTLA-4 have been evaluated in several tumor types (4), which increased response rates but also the frequency and severity of immune related adverse events (5, 6).

It has been proposed that the difference between CI-responsive or “hot” tumors i.e., melanoma and NSCLC and CI-non-responsive or “cold” tumors i.e., pancreas, prostate, ovarian and microsatellite stable (MSS)-colorectal is the density of T cell infiltration; the denser the immune infiltrate (i.e., the hotter the tumor) the greater the likelihood of benefit from checkpoint blockade. Conversely, the less dense the infiltrate (i.e., the colder or the more “immune excluded”) the tumor the less responsive to checkpoint blockade. The positive effect of tumor infiltrating lymphocytes (TILs) has been reported in several cancers (7–9).

Inefficient trafficking of T cells to the tumor is a function of several factors including an aberrant vasculature with irregular blood flow (10) and the presence of stromal cells, the most abundant of which are tumor associated macrophages (TAMs) that elaborate an anti-inflammatory immune-regulatory agenda rather than a pro-inflammatory and phagocytic one (11) Anti-tumor macrophages are referred to as M1 while pro-tumor macrophages are referred to as M2 (12). Upregulation of CD47, a checkpoint (13) for innate immunity that is ubiquitously present on phagocytic cells, including monocytes, macrophages, dendritic cells and neutrophils, promotes M2 polarization; hence, CD47 inhibition or downregulation is associated with immune sensitization (14).

RRx-001 is a minimally toxic NLRP3 inhibitor (15, 16) in Phase 3 for small cell lung cancer (SCLC) (17) that is associated with vascular normalization (18) properties as well as epigenetic inhibition and tumor associated macrophage repolarization through CD47 downregulation (19, 20). The interaction of CD47 with its ligand signal regulatory protein-α (SIRPα) serves as a marker of self to innate immune cells like macrophages, which engulf foreign cells but not self, and, in this way, CD47 expression protects cancer cells from phagocytic clearance (21). The upregulation of CD47 expression is generally epigenetic (22), and recent unpublished evidence has demonstrated that RRx-001-mediated downregulation of CD47 is related to epigenetic inhibition. Unlike traditional CD47 antibody inhibitors (23), RRx-001 is not associated preclinically or clinically with anemia, thrombocytopenia (24) or any immune-related adverse events. Moreover, to date, in over 300 patients treated no dose limiting toxicities (DLTs) have been observed with RRx-001 and no maximally tolerated dose (MTD) has been reached (25).

Given the favorable toxicity profile of RRx-001 and since both CD-47 and PD-L1 serve as innate and adaptive checkpoints, respectively, it was hypothesized that the combination of RRx-001 and nivolumab would be well-tolerated and potentially active in tumor types that were traditionally non-CI-responsive due to the broadness of induced innate and adaptive immune stimulation against target tumors.

Different dosing schedules exist for the treatment with RRx-001, ranging from pretreatment as a single agent once or twice weekly for up to 1 month or to co-administration with a combination partner every week or every other week until progression. Schedules, which involve pretreatment may be less active in combination with immunotherapy since RRx-001 administration has been associated with the generation of the immunosuppressive and pro-fibrotic cytokine, TGF-β (26). In this study, escalating doses of RRx-001 were administered once weekly with 12 ccs of autologous blood in combination with nivolumab 3 mg/kg every other week in previously treated patients with advanced cancer and no remaining therapeutic options.

The top dose of RRx-001 chosen was 16 mg since activity has been observed at approximately this dose in the Phase 1 study (27) and higher doses of RRx-001 have been associated with more pseudoprogression (28), a potential concern in combination with nivolumab since checkpoint inhibitors also induce pseudoprogression secondary to the presence of inflammatory infiltrate and necrosis (29).

No biopsies were performed, and no biomarkers were analyzed or assessed before or during this study. In another study (ClinicalTrials.gov Identifier: NCT02489903), an association was observed between tumor associated macrophage (TAM) density and response to RRx-001. However, as no biopsies were performed in PRIMETIME due to investigator and patient reluctance, this correlation could not be confirmed (30).

Commonly used biomarkers to predict response to checkpoint inhibitors include tumoral PD-L1 expression and presence of high tumor mutation burden (TMB), deficient mismatch repair (dMMR)/high microsatellite instability (MSI-H); however, the PD-L1, TMB, and dMMR/MSI-H status of these patients was and is unknown. On-treatment “liquid biopsy”, that is circulating tumor DNA (ctDNA) analysis may provide early evidence of response and progression both to RRx-001 and checkpoint inhibitors, but this was not performed.

One potential biomarker of efficacy, which it is possible to assess, is that of pseudoprogression wherein tumors, having initially enlarged due to immune cell infiltration, subsequently regress or stabilize as these immune cells eliminate cancer cells (31). Pseudoprogression has been widely seen in the context of RRx-001 administration both clinically and preclinically, and usually correlates with benefit (17, 28, 32).

## Materials and methods

### Study design

This was a phase 1 single-center, open-label, single-arm, dose escalation study. There were four predefined dose levels of RRx-001: 2, 4, 8 and 16 mg while the dose of nivolumab remained fixed at 3 mg/kg given once every other week. A 3 + 3 phase 1 design was used for enrollment. Dose-limiting toxicity was defined as a combination treatment-related ≥ grade 3 toxicity according to the National Cancer Institute Common Terminology Criteria for Adverse Events (CTCAE) V.4.03 not reversible to grade 2 or less within 96 hours. The protocol predefined maximum dose level to be investigated was 16 mg.

### Study population

Patients with solid tumors with progression of their disease following standard of care were included.

Key eligibility criteria verified during the screen procedures were age ≥18 years; ECOG performance status of 0, 1 or 2; normal hematological, liver and renal function tests; and no history of autoimmune disease. Treatment was administrated on an outpatient basis.

### Adverse events

Adverse events were graded according to the CTCAE V.4.03. Toxicity was assessed on each day of treatment and weekly in between treatments. A complete blood count with differential and platelets and metabolic panel were repeated weekly during treatment.

### Study procedures

Objective tumor responses were assessed every six weeks after initiation of study treatment and tumor response was assessed according to the Response Evaluation Criteria in Solid Tumors (RECIST) V.1.1 criteria.

### Study objective

The primary objective was to evaluate the safety of escalating doses of RRx-001 in combination with nivolumab. The secondary objective was the best objective tumor response per RECIST at 12 weeks.

### Statistical analyses

Overall objective tumor response (ORR) per RECIST criteria was summarized for the intention-to-treat (ITT) analysis set comprising all enrolled patients who received study drug dose (partial or complete, n=12).

ORR response was defined as the percentage of patients experiencing a complete response or a partial response (RECIST). Clinical benefit (disease control) was defined as CR/PR or stable disease (SD). Unevaluable patients were counted as well as non- responders.

Two-sided 95% confidence intervals for categorical variables were derived according to Clopper-Pearson formula.

## Results

### Patients’ baseline characteristics

Between January 2015 and November 2015, a total of 12 patients were screened and 12 eligible patients started study treatment. The median age was 56 (range 29.5–78.5). 83% of patients had an ECOG score of 1. The primary tumor types included two breast, two esophageal, one adenoid cystic carcinoma, one ovarian, one renal cell, one metastatic sarcoma, one uterine, one cervical and one metastatic osteosarcoma. All patients had previously progressed on standard of care. See **Table 1**. Only one patient with renal cell carcinoma had been pretreated with immunotherapy, ipilimumab.

**Table 1:** Baseline characteristics of patients.

Age	N	Mean	Median	Standard Deviation (sd)	Min	Max	Range
	12	57.0	56.0	13.2	29.5	78.5	49.0
**Sex**	**Status**	**N**	**%**	**Cumulative %**			
	F	8	66.7	66.7			
	M	4	33.3	100.0			
	Total	12	100.0	100.0			
**ECOG**	**Status**	**N**	**%**	**Cumulative %**			
	0	1	8.3	8.3			
	1	10	83.3	91.7			
	2	1	8.3	100.0			
	Total	12	100	100.0			
**Race**	**Status**	**N**	**%**	**Cumulative %**			
	Black	6	50.0	50.0			
	White	1	8.3	58.3			
	Hispanic	5	41.7	100.0			
	Total	12	100.0	100.0			
**Tumor Type**	**Status**	**N**	**%**	**Cumulative %**			
	Adenoid cystic carcinoma	1	8.3	8.3			
	Breast	2	16.7	25.0			
	Cervical	1	8.3	33.3			
	Esophageal	2	16.7	50.0			
	Metastatic pleiomorphic sarcoma	1	8.3	58.3			
	Metastatic osteosarcoma	1	8.3	66.7			
	Ovarian	1	8.3	75.0			
	Renal cell	1	8.3	83.3			
	Uterine	2	16.7	100.0			
	Total	12	100.0	100.0			

### Dose level of RRx-001 and number of patients

Across all four dose levels, treatment was generally well tolerated (**Table 2**). Higher doses did not lead to a higher incidence of adverse events. Only one patient with nivolumab discontinued study treatment because of an adverse event. Three ≥ 3 adverse events, tumor pain due to pseudoprogression, were deemed related to the combination by the clinical site. Infusion-related pain (33.3%) was the main adverse event attributed to RRx-001.

**Table 2:** All treatment emergent adverse events.

Adverse Event	Incidence RRx-001 plus Nivolumab (N=12) n (%)	Grade	Dose
Fatigue	66.7 (10/12)	2	2
Gait disturbance	8.3 (1/12)	2	4
Gait disturbance	8.3 (1/12)	3	16
Edema	16.7 (2/12)	1	8
Cyst	8.3 (1/12)	1	2
Peripheral edema	8.3 (1/12)	2	2
Back pain	8.3 (1/12)	2	2
Back pain	8.3 (1/12)	3	2
Myalgia	16.7 (2/12)	1	2
Arthralgia	16.7 (2/12)	1	2
Bone pain	8.3 (1/12)	2	2
Flank pain	8.3 (1/12)	2	2
Groin pain	8.3 (1/12)	1	2
Musculoskeletal pain	8.3 (1/12)	1	8
Pain in extremity	8.3 (1/12)	3	16
Pain in jaw	8.3 (1/12)	1	4
Constipation	41.7 (5/12)	1	4
Abdominal pain	16.7 (2/12)	1	4
Diarrhea	16.7 (2/12)	1	4 and 8
Anal incontinence	8.3 (1/12)	2	8
Nausea	8.3 (1/12)	1	16
Vomiting	8.3 (1/12)	2	16
Decreased appetite	58.3 (7/12)	1	16
Hyperglycemia	8.3 (1/12)	2	16
Hyperglycemia	16.7 (2/12)	3	16
Cachexia	16.7 (2/12)	2	16
Cough	33.3 (4/12)	1	2
Dyspnea	8.3 (1/12)	1	2
Dyspnea	16.7 (2/12)	3	4
Hemoptysis	16.7 (2/12)	2	4
Bronchiectasis	8.3 (1/12)	3	4
Pleuritic pain	8.3 (1/12)	2	4
Pneumonitis	8.3 (1/12)	3	4
Pneumothorax	8.3 (1/12)	3	4
Pulmonary embolism	8.3 (1/12)	3	8
Pulmonary hypertension	8.3 (1/12)	3	8
Wheezing	8.3 (1/12)	1	4
Anemia	50 (6/12)	2	2
Bacteremia	8.3 (1/12)	3	4
Pneumonia	8.3 (1/12)	3	4
Post procedural infection	8.3 (1/12)	2	2
Rhinitis	8.3 (1/12)	1	8
Sepsis	8.3 (1/12)	3	4
Upper respiratory	8.3 (1/12)	1	2
Urinary tract infection	8.3 (1/12)	2	8
Infusion related reaction	33.3 (4/12)	2	All dose levels
Seroma	8.3 (1/12)	4	2
Tumor pain	8.3 (1/12)	3	2
Tumor hemorrhage	8.3 (1/12)	3	2
Peripheral neuropathy	33.3 (4/12)	1	2 and 4
Headache	16.7 (2/12)	1	4
Dysgeusia	8.3 (1/12)	1	4
Facial paralysis	8.3 (1/12)	1	4
Sciatica	8.3 (1/12)	1	2
Alopecia	16.7 (2/12)	1	8
Ecchymosis	16.7 (2/12)	1	2
Night sweats	8.3 (1/12)	1	4
Rash	8.3 (1/12)	1	4
Skin hyperpigmentation	8.3 (1/12)	1	8
Insomnia	8.3 (1/12)	2	2
Anxiety	8.3 (1/12)	2	2
Depression	8.3 (1/12)	2	2
Hyperthyroidism	8.3 (1/12)	1	2
Hypothyroidism	8.3 (1/12)	2	4
Inappropriate antidiuretic hormone secretion	8.3 (1/12)	3	4
Decreased weight	16.7 (2/12)	2	2 and 4
Vaginal hemorrhage	16.7 (2/12)	2	4
Pelvic pain	8.3 (1/12)	2	4
Vaginal discharge	8.3 (1/12)	2	8
Tinnitus	8.3 (1/12)	1	8
Diplopia	8.3 (1/12)	1	8
Chronic kidney disease	8.3 (1/12)	3	4
Deep vein thrombosis	8.3 (1/12)	2	4

### Antitumor activity

**Figure 1:**
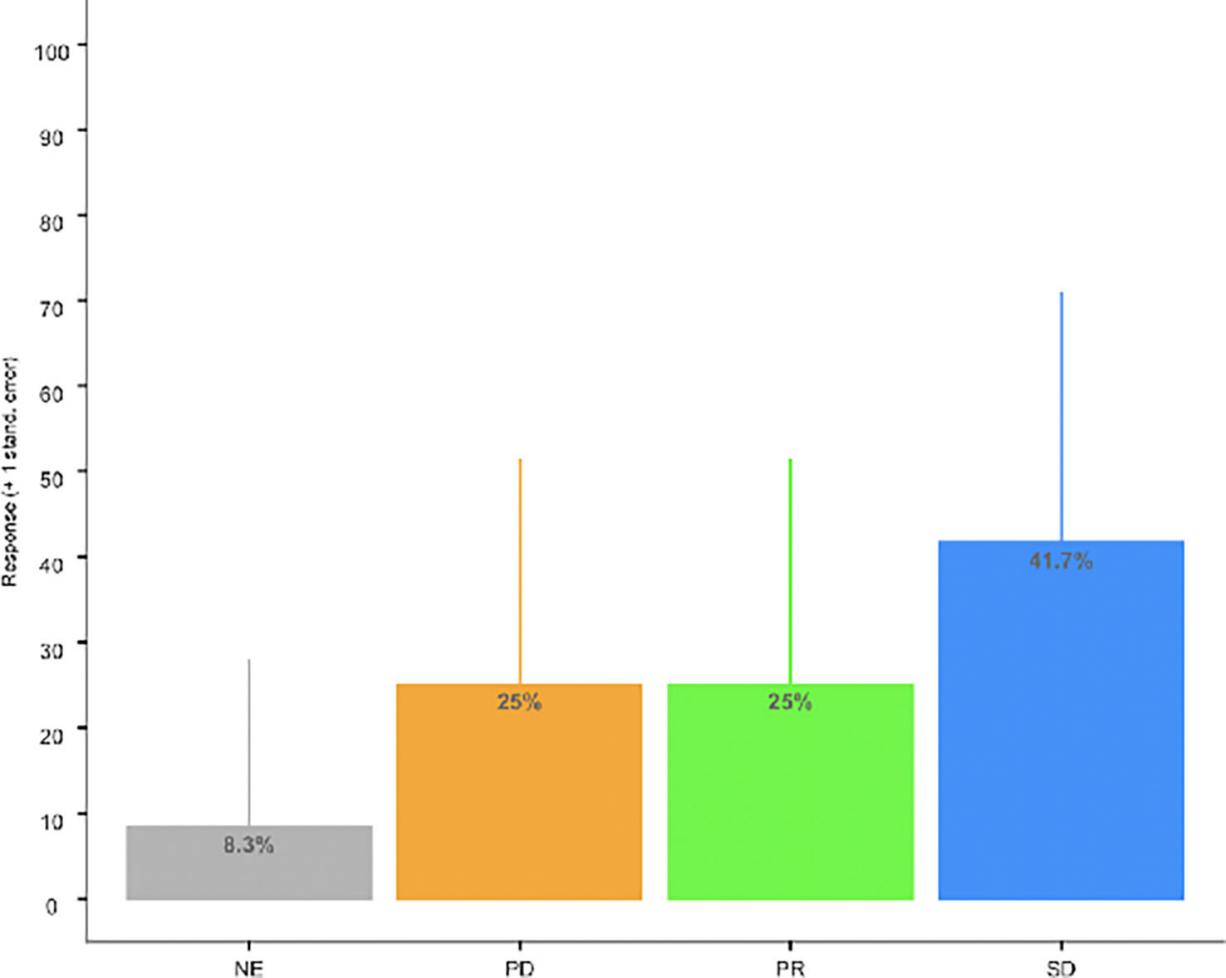
Bar Chart of ORR. Disease Control Rate [DCR) (SD + PR: 66.7% (95% CI: 34.9, 90.1)].

All patients but one was evaluable for tumor response. Three unconfirmed objective tumor responses were observed at 12 weeks. Five patients achieved stable disease at 12 weeks for a DCR of 67% (95% CI: 34.9, 90.1). See **Figure 1** and **Table 3**. The median progression-free and overall survival for the whole study population were not calculated due to incomplete follow up.

**Table 3:** Overall response rate at 12 weeks.

Disease Status	Sample Size	Response (%)	Total Sample Size
Progressive Disease (PD)	3	25.0	12
Partial Response (PR)	3	25.0	12
Stable Disease (SD)	5	41.7	12
Clinical Benefit (PR or SD)	8	66.7	12
Unevaluable	1	8.3	12

### Correlation of response by dose level analysis

To determine whether the observed effects are related to dose of RRx-001 when combined with PD-1 blockade, a Spearman product-moment correlation analysis between response (converted to a categorized variable where 1 = PR, 0 (= SD or PD or NE) is carried out, and the resulting correlation estimate is approximately 12.6% (p = 0.6973, Spearman product-moment test, an insignificant result). The limitations of the sample size in this pilot study makes the Spearman correlation analysis results a challenge to interpret, and a future study to test the reliability of such analyses is necessary.

### Duration of response analysis

**Figure 2:**
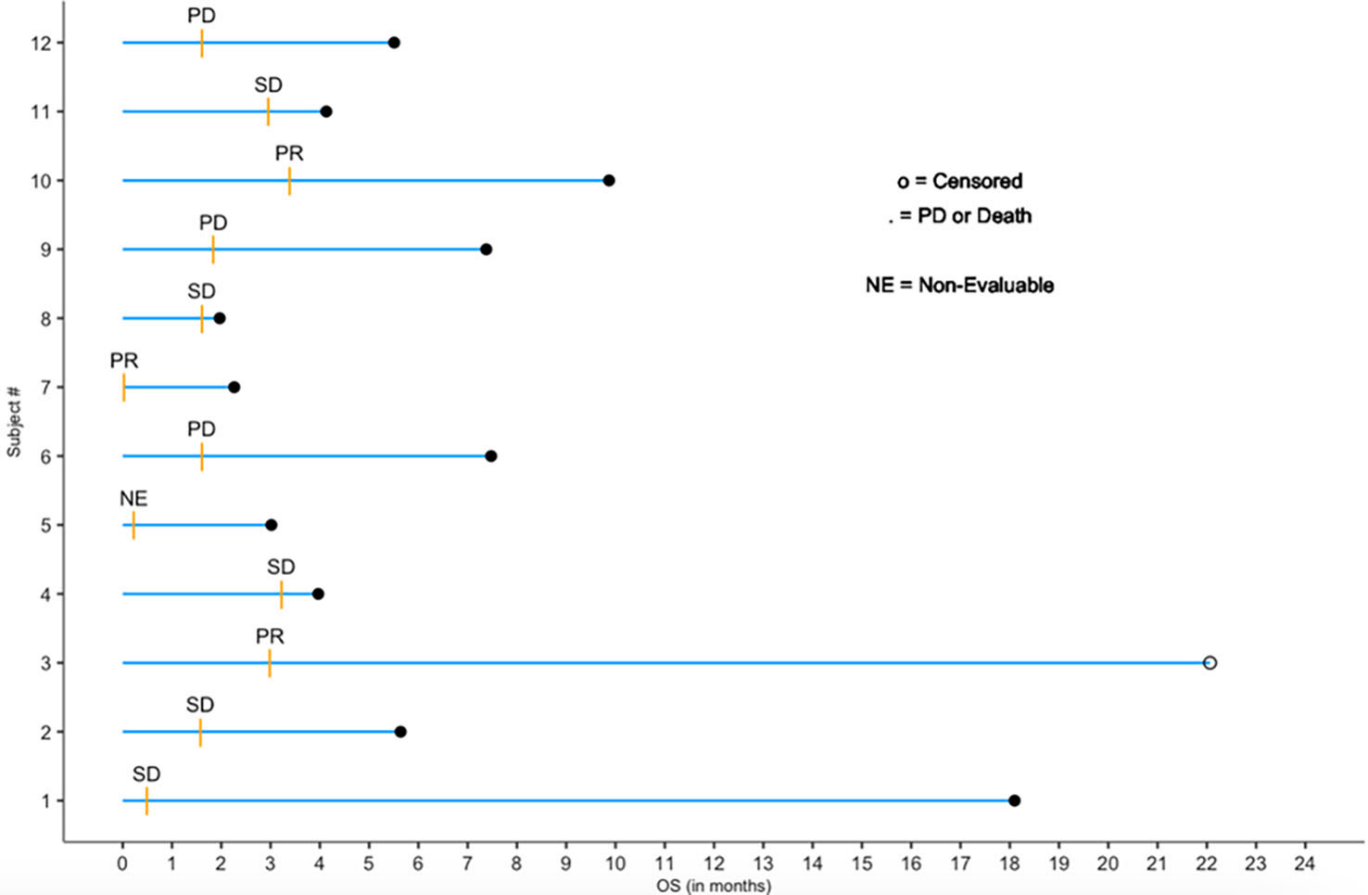
Follow-up swimmer plot by exposure months and response status.

Patients were followed beyond 12 weeks, and the follow up gathered information comprises safety and overall survival data. Defining duration of response (DoR) as the time from the first tumor response (CR or PR) to the first subsequent time of progression (or death), **Figure 2**, below, depicts a survival swimmer plot annotated using tumor response status (CR, PR, SD, PD or NE (= non-evaluable)). Since there were 3 responders (PR, unconfirmed, out of 12 patients) with DoR = 2.2, 6.5 months, and a 19.1 month-censored DoR, a median DoR *via* the standard Kaplan-Meier analysis is not suitable for a reliable estimate given the sample size limitation of this study.

## Discussion

In this small pilot phase 1 study with mainly non-checkpoint inhibitor responsive tumors, dose escalation of RRx-001 (bromonitrozidine) in combination with fixed dose nivolumab for up to 12 weeks was well-tolerated and no treatment-limiting toxicity was encountered. One patient with pre-existing lung disease developed mild pneumonitis, which led to her discontinuation from the trial. Pneumonitis was not designated as a DLT since pre-existing lung disease is thought to be a risk factor for its development (33) and, in addition, pneumonitis is a known toxicity of nivolumab (34). Likewise, two patients were hospitalized for pseudoprogression (pain due to tumor enlargement), which was deemed to be related to the combination of RRx-001 and nivolumab. However, since pseudoprogression is thought to be a favorable prognostic sign and may, in fact, serve as a biomarker of efficacy, especially since one of these patients, Subject 1, developed durable stable disease (≥ 17 months) and the other one, Subject 2, was stable for >4 months, it was not designated as a DLT.

A maximally tolerated dose was not established within the narrow dose range explored in the phase 1 trial. According to the protocol, it was the intention to dose the combination indefinitely until progression but, unfortunately, this was not possible due to the unavailability of nivolumab.

Three unconfirmed partial responses (PRs) were observed in renal cell, cervical and esophageal cancer. Renal cell is, historically, known to be one of the tumor types where checkpoint inhibitors are effective. Previously this patient received IL-2 but did not respond so it is possible that RRx-001 contributed to the benefit that she experienced. By contrast, responses in cervical and esophageal cancer are atypical; however, the microsatellite status of these tumors is unknown, which would significantly influence the likelihood of response, since microsatellite instable-high, (MSI-H), tumors are known to be exceptionally sensitive to therapy with PD-1 immune checkpoint inhibitors (35). The disease control rate (DCR) was 66.7% (8/12), which also may suggest benefit for the combination although, given the small sample size, it is equally possible that chance alone was responsible. One patient with sarcoma was deemed non-evaluable for response because he voluntarily discontinued treatment and refused any further intervention after unsuccessful drainage of a recurrent seroma.

These observations deserve confirmation in a larger sample set to determine whether the combination of RRx-001 and nivolumab (or another checkpoint inhibitor) can turn cold tumors hot i.e., to generate anti-tumor immune responses in non-immunogenic tumors and for how long.

## Conclusion

In patients with pretreated advanced solid tumors, the combination of nivolumab and dose-escalated RRx-001 is safe and well-tolerated. The unconfirmed ORR and DCR are suggestive of benefit and provide a basis for further study of this regimen to determine whether it may sensitize nonimmunogenic tumors or tumors refractory to immunotherapeutic agents.

## Data availability statement

The raw data supporting the conclusions of this article will be made available by the authors, without undue reservation.

## Ethics statement

The studies involving human participants were reviewed and approved by The Geneva Foundation. The patients/participants provided their written informed consent to participate in this study.

## Author contributions

TR, BO, and NA conceived and wrote the manuscript SC, MQ, JW, and PC reviewed and edited. All authors contributed to the article and approved the submitted version.
